# Semi-Automatic Quantification of Subsolid Pulmonary Nodules: Comparison with Manual Measurements

**DOI:** 10.1371/journal.pone.0080249

**Published:** 2013-11-21

**Authors:** Ernst Th. Scholten, Bartjan de Hoop, Colin Jacobs, Saskia van Amelsvoort-van de Vorst, Rob J. van Klaveren, Matthijs Oudkerk, Rozemarijn Vliegenthart, Harry J. de Koning, Carlijn M. van der Aalst, Willem Th M. Mali, Hester A. Gietema, Mathias Prokop, Bram van Ginneken, Pim A. de Jong

**Affiliations:** 1 Department of Radiology, University Medical Centre Utrecht, Utrecht, The Netherlands; 2 Department of Radiology, Kennemer Gasthuis, Haarlem, The Netherlands; 3 Image Analysis Group, Department of Radiology UMC St Radboud, Nijmegen, The Netherlands; 4 Fraunhofer MEVIS, Bremen, Germany; 5 Departement of Pulmonology, Lievensberg Hospital, Bergen op Zoom, The Netherlands; 6 Department of Radiology, University Medical Centre, Groningen, The Netherlands; 7 Department of Public Health, Erasmus Medical Centre, Rotterdam, The Netherlands; 8 Department of Radiology, UMC St Radboud, Nijmegen, The Netherlands; University of Navarra, Spain

## Abstract

**Rationale:**

Accurate measurement of subsolid pulmonary nodules (SSN) is becoming increasingly important in the management of these nodules. SSNs were previously quantified with time-consuming manual measurements. The aim of the present study is to test the feasibility of semi-automatic SSNs measurements and to compare the results to the manual measurements.

**Methods:**

In 33 lung cancer screening participants with 33 SSNs, the nodules were previously quantified by two observers manually. In the present study two observers quantified these nodules by using semi-automated nodule volumetry software. Nodules were quantified for effective diameter, volume and mass. The manual and semi-automatic measurements were compared using Bland-Altman plots and paired T tests. Observer agreement was calculated as an intraclass correlation coefficient. Data are presented as mean (SD).

**Results:**

Semi-automated measurements were feasible in all 33 nodules. Nodule diameter, volume and mass were 11.2 (3.3) mm, 935 (691) ml and 379 (311) milligrams for observer 1 and 11.1 (3.7) mm, 986 (797) ml and 399 (344) milligrams for observer 2, respectively. Agreement between observers and within observer 1 for the semi-automatic measurements was good with an intraclass correlation coefficient >0.89. For observer 1 and observer 2, measured diameter was 8.8% and 10.3% larger (p<0.001), measured volume was 24.3% and 26.5% larger (p<0.001) and measured mass was 10.6% and 12.0% larger (p<0.001) with the semi-automatic program compared to the manual measurements.

**Conclusion:**

Semi-automated measurement of the diameter, volume and mass of SSNs is feasible with good observer agreement. Semi-automated measurement makes quantification of mass and volume feasible in daily practice.

## Introduction

Lung cancer screening with computed tomography (CT) has increased the awareness of a specific subtype of pulmonary nodules: the subsolid nodule (SSN). A SSN is defined as a circumscribed area of increased lung attenuation with preservation of the bronchial and vascular margins and also referred to as a ground glass opacity.[1] A SSN can be part-solid (part of the nodule completely obscures the underlying lung parenchyma) or pure nonsolid. Persistent SSNs have a high likelihood of malignancy. The ELCAP study [2] reported a malignancy rate of 34% for all nonsolid SSNs, 18% for pure ground glass lesions and 63% for part-solid SSNs. For part solid lesions even higher malignancy rates with numbers up to 75% are reported. [3]

Recently, a statement from the Fleischner Society with recommendations for the management of SSNs detected at CT was published.[4] It was recommended that, because most non solid SSNs prove either to be benign or pre malignant,a 3 month and then annual follow up is appropriate. A monitoring strategy can obviate unnecessary surgery and potentially avoid overdiagnosis in cases in which no change is identified. Monitoring should also allow early identification of lesions that will prove to be adenocarcinomas manifesting as pure nonsolid SSNs. For solitary part-solid SSNs, especially those in which the solid component is larger than 5 mm, it was stated that, when persistent, these should be considered malignant until proven otherwise. It is evident that accurate automated measurements of SSNs can be valuable to follow these recommendations and detect (absence) of growth as early as possible. For that purpose volume measurements are preferable to diameter measurements. For SSNs there is evidence that mass measurements are preferable to volume measurements.[5] However, most volumetry software is developed for solid pulmonary nodules and fails when segmenting SSNs. SSNs were manually segmented previously by two observers which took about 10 minutes for each nodule, making it inappropriate for daily routine.[5] Recently, software has become available for semi-automatic segmentation of SSNs in which these nodules are segmented within a second.[6], [7]

The aim of this study was to the test the feasibility of nodule volumetry software for SSNs and to compare diameter, volume and mass measurements on CT exams of subsolid pulmonary nodules of the semi-automated software to the results of the manual segmentations.

## Methods

### Study Participants

This is an ancillary study of the Dutch-Belgian Lung Cancer Screening Trial (NELSON trial; ISRCTN63545820). The NELSON trial was approved by the Dutch and Belgian Ministries of Health and by the ethical review board of the participating hospitals. Written informed consent was obtained from each participant. The trial population comprised current or former smokers between 50 and 75 years old at time of inclusion with a smoking history of at >15 cigarettes/day during >25 years or >10 cigarettes/day during >30 years. Former smokers were included only if they quit smoking ≤10 years before the start of the study. Exclusion criteria for participating in the lung cancer screening trial were self-reported moderate or poor health status and/or inability to climb two flights of stairs, recent chest CT, current or previous history of cancer at time of inclusion, and body weight ≥140 kg. Participants were randomized to the screening arm (screening with low-dose CT) or the control arm (no imaging).

For the current analysis, all 2994 baseline CT examinations performed at one of the study sites (University Medical Center, Utrecht, the Netherlands) were included. All 33 SSNs > 5 mm detected in 33 volunteers and recorded in the Nelson Management System were used in our current evaluation.

### CT Scanning and reading protocol

The NELSON protocol included a low-dose CT-examination. Patients were imaged using a 16-detector-row CT scanner (Mx8000 IDT or Brilliance-16, Philips Medical Systems, Cleveland, OH) in helical mode with 16×0.75-mm collimation and 15-mm table feed per rotation (pitch 1.3). CT acquisition was done in full inspiration. No intravenous contrast was injected. Exposure settings were 30mAs at 120kVp for patients weighing <80 kg, and 30mAs at 140kVp for those weighing more than 80 kg. Axial images of 1.0 mm thickness were reconstructed at 0.7 mm increment with a 512×512 matrix, using a moderately soft kernel and the smallest field of view (FOV) that included both lungs. The CT exams were evaluated by double reading with a consensus reading in case of discrepant results. All CT exams were read for nodules and detected nodules were characterized as solid nodule or subsolid nodule, either nonsolid or part-solid.

### Subsolid nodule evaluation and measurements: detected SSNs

Of the 33 participants with a GGN 25 (76%) were male. The age was 61.5 (SD 6.4) years, the number of packyears was 41.1 (SD 19.0) and 17 (51.5%) were current smokers All included SSNs were manually segmented previously by two observers.(BdH,SvdV) These results and the agreement between the observers have previously been published.[5]

For the present study a software program was used to semi-automatically segment and quantify the nodule. Two observers, one with 10 years experience with chest CT (PdJ) and one with over 30 years experience (ETS) measured the nodules independently with this software program. One observer (ETS) repeated the measurements after >1 month to assess intraobserver agreement.

The prototype of this software program has been described previously [6] and was adapted to handle SSNs. The user can either click a center point or draw a stroke on the largest diameter of the nodule as an input to the algorithm. Based on this user input, a volume of interest (VOI) is automatically defined around the nodule. An initial segmentation is acquired by region growing using thresholds applicable to subsolid nodules. Default value for the lower threshold is −750 HU, and for the higher threshold −150 HU. Two parameters, density threshold value and roundness versus irregularity, can be adjusted by the user of the program to optimize the segmentation if this is felt to be necessary by the observer.

Finally, a sequence of morphological operations is used to remove the chest wall and adjacent vessels, if applicable.

SSN mass was calculated by expressing attenuation values in terms of physical density. CT attenuation in Hounsfield units can be translated directly into physical density in milligrams per milliliter by adding 1000 to the Hounsfield unit value. For soft-tissue nodules, the prerequisites for this approach are that the nodule contains no calcium and that no contrast material was injected. The mass within the nodule volume, as outlined on all sections that contained the nodule, was calculated by multiplying nodule volume by mean nodule density (ie, mean CT number + 1000) [8].

### Data analysis

Data are presented as mean and standard deviation (SD) or percentage (%). Diameter, volume and mass of the SSNs were compared between the mean of the two results of the manual measurement and the various semi-automatic measurements using the method described by Bland and Altman [9] and by paired T-tests. Observer agreement was calculated as an intraclass correlation coefficient. P-values <0.05 were considered significant.

## Results

### Subjects and nodule characteristics

Thirty-three participants had a total of 33 SSNs at baseline according to NMS. The semi-automated program failed in none of the nodules. Of these 33 subsolid nodules, 19 were pure ground glass and 14 were part-solid. The mean diameter was just above one centimetre (Table 1). Observer 1 did not make any adjustment to the semi-automated measurements in 22 (67%) of the measurements, in 7 (21%) the density cut-off was adjusted and in 4 (12%) the roundness was adjusted. Observer 2 did not make any adjustment in 24 (73%) of the measurements and the density threshold was adjusted in 9 (28%) cases. Manual measurements involved about 10 minutes per SSN and semi-automatic measurements involved a few seconds.

**Table 1:** Results of manual and semi-automatic measurements of 33 subsolid nodules on CT

	Semi-automatic measurements		Manual measurement		Difference
	mean	SD	Mean	SD	p-value
**Observer 1 first**					
Diameter (mm)	11.2	3.3	12.2	3.7	<0.001
Volume (ml)	935	692	1201	934	<0.001
Mass (milligrams)	379	311	431	369	0.007
**Observer 1 second**					
Diameter (mm)	10.8	3.8	12.2	3.7	<0.001
Volume (ml)	930	809	1201	934	0.001
Mass (milligrams)	378	341	431	369	0.02
**Observer 2**					
Diameter (mm)	11.1	3.7	12.2	3.7	<0.001
Volume (ml)	985	797	1201	934	0.001
Mass (milligrams)	399	344	431	369	0.07

Differences are tested with the paired samples T test.

### Comparison of manual and semi-automatic measurements and observer agreement

**Figure 1:**
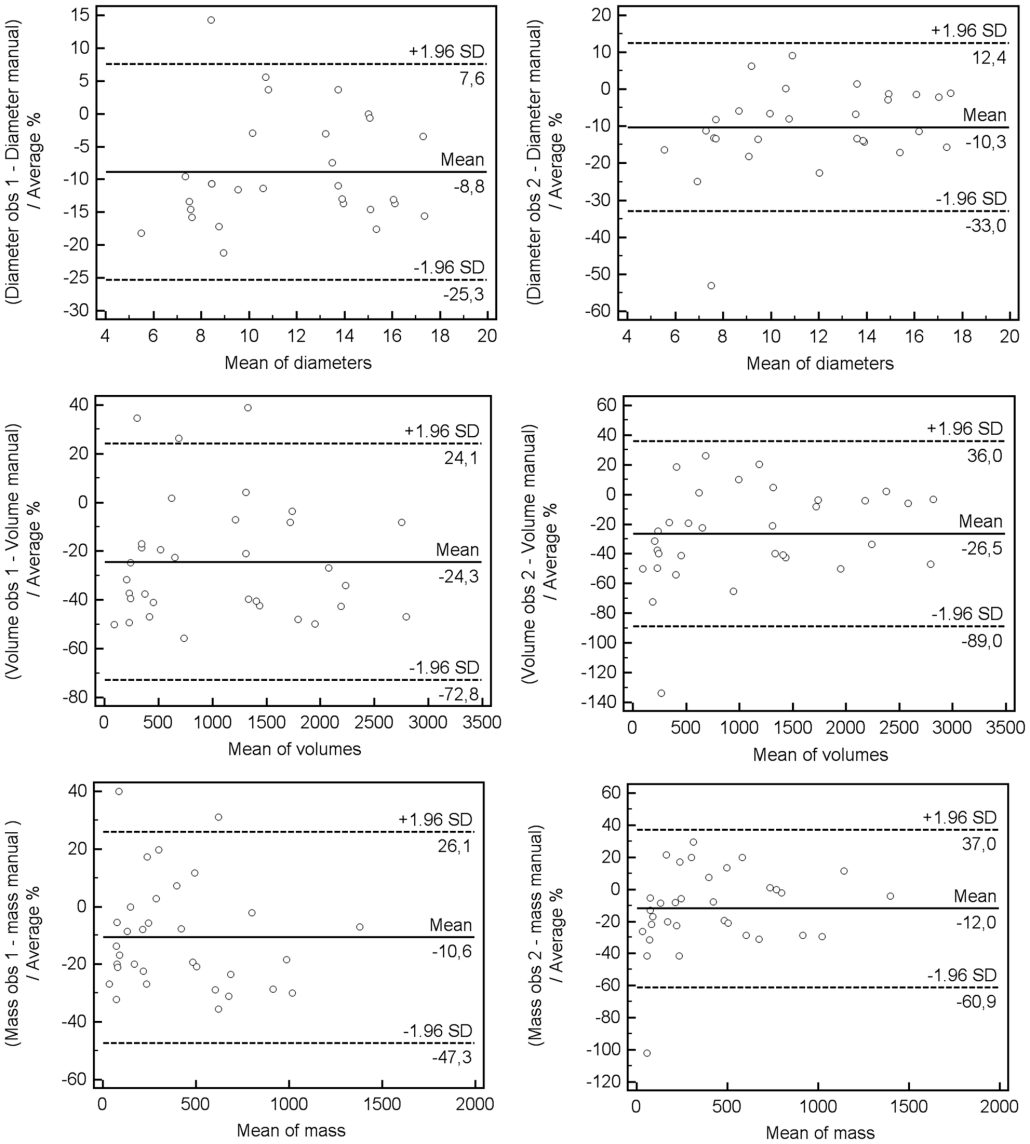
Bland-Altman plots for two observers of relative differences between manual and semi-automatic measurements of subsolid nodules on CT for diameter (top row), volume (middle row) and mass (bottom row).

Nodule diameter, mass and volume were significantly different between the mean values of the two manual measurements and semi-automated measurements (all p<0.05), except for the mass measurements performed by observer 2 (Table 1). The average difference between manual and the three semi-automated measurements was in diameter 1.0 to 1.4 mm, in volume 215 to 271 ml and in mass 32 to 52 mg. On average diameter, volume and mass were measured 8.8%−10.3%, 24.3%−26.5% and 10.6%−12.0% smaller with the manual measurements when compared to the semi-automated measurements (Figure 1). The intraclass correlation between and within observers was good (>0.89, Table 2) and the agreement was best for mass measurements.

**Table 2:** Between and within observer agreement for semi-automatic subsolid nodule measurements.

	Diameter (mm)	Volume (ml)	Mass (milligrams)
Observer 1, first reading	0.89	0.92	0.95
Versus			
Observer 1, second reading			
Observer 1, first reading	0.95	0.94	0.97
versus Observer 2, reading			
Observer 1, second reading	0.90	0.92	0.95
Versus			
Observer 2			

Data given are intraclass correlation coefficients.

## Discussion

Our results show that semi-automated measurement of SSNs is feasible with good observer agreement. This result is in agreement with the previous conclusions of Oda et al [10] who concluded that with computer-aided volumetry of subsolid nodules, the relative volume measurement error was small for nodules 5 mm in diameter or larger. Intraobserver and interobserver agreement was relatively good for nodules 8 mm in diameter or larger.

SSN dimensions measured by semi-automated volumetry software compared closely to the results of manual measurements, but dimensions were slightly larger. These results are promising as the importance of SSN quantification is increasingly recognized and manual measurements are impractical due to the time involved in the segmentation.

SSNs are a major challenge, both clinically [11] and in a lung cancer screening setting [2], because these nodules are relatively rare, slow growing, often multiple and bear a high malignancy rate. Although transient SSNs can represent a large range of benign diseases, persistent SSNs have a high likelihood of malignancy, with reported malignancy rates ranging from 18% to 75% [2], [3]). In the first round of the Dutch-Belgian lung cancer screening trial only 2.0% of the detected nodules, of the total of 8673 nodules found in 7557 participants, were nonsolid nodules or part-solid nodules[12]. In the NELSON trial volumetry software was used to measure dimensions of solid nodules, because volumetry has been proven to be superior to diameter measurements in terms of accuracy and reproducibility. The SSN were assessed visually and by manual diameter measurement as no volumetry software was available for SSNs and volumetry software for solid nodules often failed when segmenting SSNs. However, for SSN accurate measurement is becoming increasingly important, too, as differentiation between benign and malignant nodules is largely based on change in size or on the development of a solid component. Recently, a statement from the Fleischner Society with recommendations for the management of SSNs detected at CT was published.[4] For persistent solitary nonsolid nodules it was recommended that monitoring is appropriate to enable early detection of even subtle interval change in their appearance. Such monitoring could prevent overtreatment and allow early identification of growing lesions that prove to be adenocarcinomas. For solitary part-solid SSNs, especially those in which the solid component is larger than 5 mm, it was stated that these should be considered malignant until proven otherwise provided no regression of the nodule is seen at a follow-up CT examination performed in 3 months. It is evident that accurate semi-automated measurements of SSNs can be valuable to follow these recommendations and detect growth as early as possible. Furthermore, for SSNs there is evidence that mass measurements are preferable to volume and diameter measurements.[5]

In order to be feasible in clinical practice segmentation of SSNs must not be time consuming but easy and swift. In a previous study, SSNs were manually segmented by two observers and it took them about 10 minutes to segment one nodule. Recently, software has become available for semi-automated segmentation of SSNs in which nodules can be segmented in a few seconds. By using the new software semi-automated measurement of SSNs were found to be feasible with good observer agreement. Reported SSN dimensions compared closely to manual measurements, but dimensions with the semi-automated approach were slightly larger than the manual measurements.

The applied semi-automated software has previously been tested in an anthropomorphic phantom study. [13] In that study the semi-automated measurements compared closely to the true values without systematic errors. It may therefore well be that the manual measurements systematically underestimate nodule dimensions. The semi-automated software can now be further tested to investigate the interscan error in the large amount of CT imaging data that has been acquired as part of lung cancer screening trials.

Our study is limited by the lack of a true ‘gold’ standard as we compared our results to those of manual segmentations. The semi-automated segmentation measured the nodules systematically larger.

**Figure 2:**
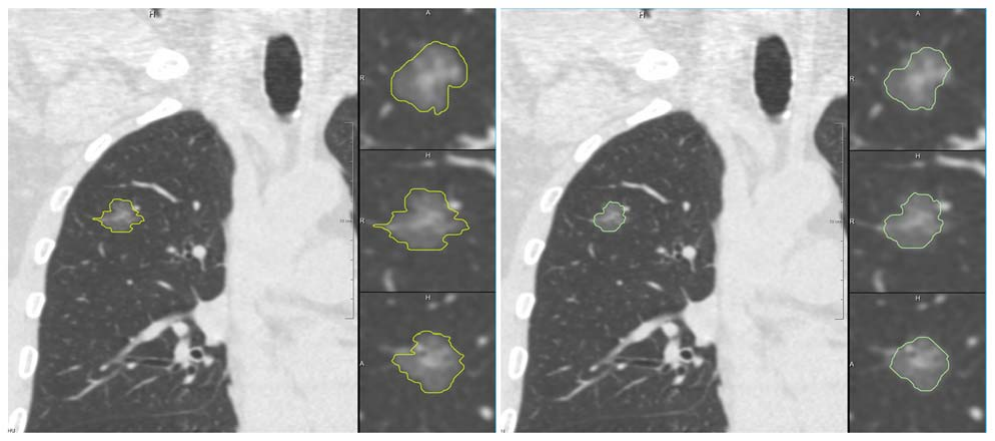
Illustration of manual and semi-automatic nodule segmentation on CT. The semi-automatic measurement is presented on the left, and the manual measurement on the right. At the right side of both images are 3 zoomed images from top to bottom axial, coronal and sagital plane. Note the irregularity of the manual measurements in the coronal and sagital plane. This is because manual segmentations are done in the axial plane, while semi-automatic measurements are truly 3D.

Our study does not provide scientific evidence that semi-automatic segmentation is more accurate than manual segmentation. However, visual inspection in the coronal and sagital planes suggests that the semi-automatic measurements were more accurate as there were skip artefacts visible in the manually drawn contours.(Fig 2) This is also supported by previous phantom studies with the semi-automatic software in which no systematic errors were found.[13].

Another limitation is the relative small number of nodules in our series. This made us refrain from analyzing even smaller subdivisions since this would not give significant results.

In conclusion, our study shows that semi-automated measurements of diameter, volume and mass of subsolid nodules are feasible with good observer agreement and without taking extra time. This makes semi-automated measurements appropriate to be used in daily clinical and screening practice.
